# Immunotherapy and radiotherapy for older patients with locally advanced rectal cancer unfit for surgery or decline surgery: a practical proposal by the International Geriatric Radiotherapy Group

**DOI:** 10.3389/fonc.2024.1325610

**Published:** 2024-02-22

**Authors:** Nam P. Nguyen, Mohammad Mohammadianpanah, Arthur SunMyint, Brandi R. Page, Vincent Vinh-Hung, Olena Gorobets, Meritxell Arenas, Thandeka Mazibuko, Huan Giap, Maria Vasileiou, Fabien Dutheil, Carmelo Tuscano, ULF Lennart Karlsson, Zineb Dahbi, Elena Natoli, Eric Li, Lyndon Kim, Joan Oboite, Eromosele Oboite, Satya Bose, Te Vuong

**Affiliations:** ^1^ Department of Radiation Oncology, Howard University, Washington, DC, United States; ^2^ Colorectal Research Center, Department of Radiation Oncology, Namazi Hospital, Shiraz University of Medical Sciences, Shiraz, Iran; ^3^ Department of Radiation Oncology, Clatterbridge Cancer Center, Liverpool, United Kingdom; ^4^ Department of Radiation Oncology, Johns Hopkins University, Bethesda, MD, United States; ^5^ Department of Radiation Oncology, Institut Bergonie, Bordeaux, France; ^6^ Department of Oral Surgery, Martinique University, Fort de France, France; ^7^ Department of Radiation Oncology, Sant Joan de Reus University, University of Rovira, I Virgili, Tarragona, Spain; ^8^ Department of Radiation Oncology, International Geriatric Radiotherapy Group, Washington, DC, United States; ^9^ Department of Radiation Oncology, Medical University of South Carolina, Charleston, SC, United States; ^10^ Department of Pharmacy, School of Health Sciences, National and Kapodistrian University of Athens, Athens, Greece; ^11^ Department of Radiation Oncology, Clinique Sainte Clotilde, Saint Denis, La Reunion, Saint Denis, France; ^12^ Department of Radiation Oncology, A.O Bianchi Melacrino, Reggio Calabria, Italy; ^13^ Department of Radiation Oncology, Mohammed VI University of Health Sciences, Casablanca, Morocco; ^14^ Department of Radiation Oncology, University of Bologna, Bologna, Italy; ^15^ Department of Pathology, Howard University, Washington, DC, United States; ^16^ Division of Neurooncology, Mt Sinai Hospital, New York, NY, United States; ^17^ Department of Radiation Oncology, Mc Gill University, Montreal, Canada

**Keywords:** older, rectal cancer, locally advanced, CPI, radiotherapy

## Abstract

The standard of care for locally advanced rectal cancer is total neoadjuvant therapy followed by surgical resection. Current evidence suggests that selected patients may be able to delay or avoid surgery without affecting survival rates if they achieve a complete clinical response (CCR). However, for older cancer patients who are too frail for surgery or decline the surgical procedure, local recurrence may lead to a deterioration of patient quality of life. Thus, for clinicians, a treatment algorithm which is well tolerated and may improve CCR in older and frail patients with rectal cancer may improve the potential for prolonged remission and potential cure. Recently, immunotherapy with check point inhibitors (CPI) is a promising treatment in selected patients with high expression of program death ligands receptor 1 (PD- L1). Radiotherapy may enhance PD-L1 expression in rectal cancer and may improve response rate to immunotherapy. We propose an algorithm combining immunotherapy and radiotherapy for older patients with locally advanced rectal cancer who are too frail for surgery or who decline surgery.

## Introduction

Rectal cancer prevalence increases significantly with age. Early screening has led to early diagnosis and improved survival. However, current recommendation for colonoscopy stops at the age of 75 even though preliminary evidence suggests that screening for colorectal cancer beyond this age limit may be beneficial (1). Thus, the disease is frequently diagnosed at a locally advanced stage and curative resection may not be possible for older patients (2). In addition, among older cancer patients who underwent surgical resection, the mortality rate increases significantly with age due to increased complication rates (3). The increased comorbidity and frailty likely contribute to the high mortality rate observed after rectal cancer surgery in older patients (4, 5). Radical rectal surgeries, whether with or without sphincter preservation, are associated with significant morbidities (6). Permanent colostomy in patients who undergo abdominoperineal resection (APR) and complications of low anterior resection (LAR) syndrome in patients who are treated with sphincter preserving surgery are very debilitating and reduce patients’ quality of life (QOL) (7). Older patients are particularly affected after due to their lower performance status prior to surgery. Thus, considering non-surgical strategies for those patients may be very valuable to maintain their QOL. As an alternative, for older rectal cancer patients who are too frail to undergo surgery or decline surgery, radiotherapy alone or combined with chemotherapy is a viable option to increase survival. However, ultimately disease progression was observed in many of them (8). Among rectal cancer patients who underwent neoadjuvant chemoradiotherapy, a complete clinical response (CCR) is frequently associated with a better prognosis and among those who adopted watchful waiting, prolonged survival has been reported (9). The preliminary report from the OPERA randomized trial is very encouraging. Among patients with clinical stage T2-T3 rectal cancer undergoing concurrent chemoradiation with capecitabine, increased CCR and organ preservation were reported among patients who had a high boost dose with contact therapy. There was no impact on survival for those who prefer watchful waiting (10). Thus, a higher radiation dose associated with chemotherapy improved CCR and also local control rates. Could CCR rates further improve with a systemic agent which is well tolerated by older and frail patients? Preliminary reports suggest that neoadjuvant chemotherapy and immunotherapy may improve CCR rates in patients with locally advanced rectal cancer (11–13). Furthermore, in a subset of patients with mismatch repair deficient (MMR-D)/microsatellite instability-high (MSI-H) rectal cancers, neoadjuvant check point inhibitors (CPI) have been reported to induce an excellent CCR rate with reduced toxicity (14–17). In this subset of patients, CPI alone may produce long-term remission and may allow the patient to avoid surgery (18). Given our recent advance in molecular biology of rectal cancer, it is possible to personalize patient treatment based on biomarkers to improve the patient quality of life (QOL).

As an organization devoted to the care of older cancer patients, minorities, and women who are frequently excluded from clinical trials, the International Geriatric Radiotherapy Group (http://www.igrg.org) would like to propose a practical protocol for older patients with locally advanced rectal cancer who are too frail to undergo surgery or who decline surgery (19). Radiotherapy and immunotherapy may induce long-term remission and potential cure in selected patients.

## Prevalence of program death ligand 1 (PD-L1) in patients with colorectal cancer

Depending on the cutoff value, the prevalence of PD-L1 ranges from 5% to 73% (20–32) in patients with colorectal cancers. This variability of PD-L1 expression across the studies is likely related to the tumor histology, grade, and stage. Non-mucinous tumor, poorly differentiated grade, and advanced stage (III, IV) are linked to a higher PD-L1 expression. High PD-L1 expression in colorectal cancer is frequently linked to a poor prognosis due to the tumor cells ability to evade the immune system (33). Program death ligand is a transmembrane glycoprotein commonly expressed on the surface of normal cells which binds to program cell death protein 1 (PD-1) present on activated T cells (34). Binding of PD-L1 to PD-1 on T cells activates the downstream signaling of these cells, thus inhibiting proliferation, cytokines production, and cytotoxicity and prevents destruction of normal cells (35). Abnormality of the PD1-PD-L1 axis has been reported to be the mechanism of autoimmune disease (36). However, cancer cells also express PD-L1 at various levels which allow them to escape killing by the immune system. Thus, preventing their binding to activated T cells by antibodies directed against PD-1, PD-L1, gene silencing, or small molecules inhibition may restore the immune system leading to tumor destruction.

Immunotherapy with CPI which is directed against PD-1 or PD-L1 has been reported to improve survival among patients with metastatic colorectal cancer (37, 38). The impact of CPI is greatest among tumors with MMR-D/MSI-H as a predictive biomarker (37). The mismatch repair system (MMR) is a highly conserved DNA repair mechanism which consists of specific DNA mismatch repair enzymes dependent on four key genes, mutL homologue 1 (MLH1), postmeiotic segregation increased 2 (PMS2), mutS homologue 2 (MSH2), and mutS 6 (MSH6). If one or more enzymes are not functional, the mismatch repair mechanism is deficient. The accumulation of errors in genetic sequences are repeated leading to high microsatellites (MSI-H). Tumors with high PD-L1 expression are frequently but not always associated with increased MMR-D/MSI-H. The rate of MSI-H among tumor with high PD-L1 expression ranges from 5 to 75%. The positivity of PD-1 and PD-L1 as well as dMMR/MSI-H are the most important predictors for response to immunotherapy in a metaanalysis of advanced colorectal cancer (39). Thus, both PD-L1 and MSI status should be investigated to assess potential tumor response to immunotherapy. **Table 1** summarizes PD-L1 prevalence in patients with colorectal cancer and its relationship to MSI-H status.

**Table 1:** Prevalence of PD-L1 expression in colorectal cancer and its relationship to MSI status.

Study	Patient No	PD-L1 expression	MSI status
Valentini et al. (20)	63	25%	75%
Peng et al. (21)	233	23.6%	NS
Choi et al. (22)	138	73.9%	75.3%
Calik et al. (23)	157	45.8%	NS
Lee et al. (24)	394	5%	63%
Li et al. (25)	632	46.9%	34.6%
Watson et al. (26)	149	7%	NS
Bertnsson et al. (27)	526	56.7%	19.9%
Rosenbaum et al. (28)	181	9%	no correlation
Chi et al. (29)	236	12.7%	30%
Ho et al. (30)	238	5.4%	5.6%
Moller et al. (31)	1800	5.1%	18.6%
Kim et al. (32)	208	12.5%	100%

PD-L1, program death ligand 1; MSI, microsatellite instability; NS, not specified.

## Modulation of PD-L1 in patients with rectal cancer

Even though PD-L1 is not a perfect biomarker for immunotherapy, high PD-L1 expression frequently correlates with response to CPI (40). Strategies to modify the tumor microenvironment in order to increase PD-L1 expression may also increase MSI-H rate.

*In vitro* experiment with colon cancer cell lines suggests that they rarely express PD-L1 on their cell membranes. However, after exposure to 5-fluorouracil (5-FU), there was a significant increase of PD-L1 expression (41). The upregulation of PD-L1 expression in tumor is mediated through the infiltration CD8 T cells in the tumor following chemotherapy to evade its destruction (42). The increase of inflammatory T cells has also been reported in clinical studies. Among seven patients with MMR-proficient (MMR-P) locally advanced rectal cancer who underwent neoadjuvant chemotherapy, there was a significant increase PD-1 positive T cells in the biopsy specimen obtained after chemotherapy compared to the one before treatment (43). Another study also corroborated the concept of chemotherapy-induced immunotherapy modulation in rectal cancer. Among 49 patients with MMR proficient (MMR-P) rectal cancer who underwent neoadjuvant chemotherapy, there was a significant increase of PD-1 positive T cells in the resected specimen compared to a control of 25 patients who had surgery alone (44). The level of PD-L1 in tumor cells were also increased in those who received neoadjuvant chemotherapy but did not achieve statistical significance. Thus, chemotherapy may make the tumor microenvironment more sensitive to immunotherapy even in MMR-P rectal cancer.

Similar *in vivo* experiments of colon cancer cells demonstrated that radiotherapy may be another effective immunomodulator. Significant increase in PD-L1 expression was observed in colonic tumor cells following radiotherapy to a total dose of, 1000 cGy in 200 cGy/fraction. Administration of CPI with radiotherapy significantly improved survival of mice injected with colonic cancer cells compared to those who had radiotherapy alone or CPI alone (45). Thus, radiotherapy acts synergistically with immunotherapy to improve local control and survival. Increased in PD-L1 expression following radiotherapy was also observed in clinical studies of rectal cancer. The expression of PD-L1 was 15% and 50% in the biopsy and resected tumor before and after radiotherapy, respectively (p=0.0005) (46). In addition, high dose radiotherapy per fraction may potentiate the effect of immunotherapy through the abscopal effect, and potentially improve survival through a reduction of distant metastases (47). As standard of care for locally advanced rectal cancer is preoperative chemoradiation, we postulate that combining those two modalities may increase further PD-L1 expression, and may lead to a better immune response.

Indeed, neoadjuvant chemoradiation has been reported to increase PD-L1 expression of the tumor cells (48–50) and the inflammatory cells in the tumor stroma (50). Even when there was no increase in tumor cells PD-L1 expression, the inflammation produced by chemotherapy has led to an increase infiltration of CD8+ T cells, and high PD-L1 expression of tumor stromal cells (T cells, B cells, dendritic cells, and macrophages) (51). The immunomodulation of chemoradiation was also corroborated in another study where not only there was an increase of the inflammatory cells in the surgical specimen but there was also a downregulation of genes regulating the MMR system leading to an alteration of MSI status (52) Thus, chemoradiotherapy or radiotherapy alone may enhance CPI effect in patients with rectal cancer. **Table 2** summarizes the potential PD-L1 upregulation by neoadjuvant treatment.

**Table 2:** Upregulation of PD-L1 following radiotherapy or chemoradiation for rectal cancer.

Study	Patient No	Treatment	PD-L1	
			Before	After
Boustani et al. (47)	74	RT alone (n=44)	15%	50%
		Chemoradiation (n=29)		
Hecht et al. (48)	199	Chemoradiation	2.1%	7.8% to 9.3%
Chiang et al. (49)	104	Chemoradiation	51%	64%
Tayshetye et al. (50)	40	Chemoradiation	10%	19%
Ogura et al. (51)	287	Chemoradiation	31.7%	49.2%

RT, radiotherapy.

Preliminary experience suggests that the combination of chemoradiation followed by immunotherapy may be beneficial to improve the response rate of neoadjuvant rectal cancer. Among 42 patients with locally advanced rectal cancer who had preoperative chemoradiation and nivolumab before surgery, pathologic complete response (pCR) was observed in 30% and 60% for those with microsatellite stable (MSS) (n=37) and MSI-H (n=5), respectively (53). Another study also corroborated the efficacy of the combined chemoradiation and immunotherapy for MMR-P/MMS rectal cancer patients (54): 23 patients underwent sequential chemoradiation and sintilimab for ultra low rectal cancer. 10 underwent surgery and had 20% pCR. Among the 13 patients who did not have surgery, 10 (76%) achieved CCR. The combined treatment was well tolerated with no death. The benefit of combining a short course of radiotherapy followed by chemotherapy and immunotherapy to improve response rate was reported in another study: 13 patients with locally advanced MMR-P rectal cancer underwent external beam radiation to a total dose of 500 cGy times 5 followed by chemotherapy and avelumab before surgery. Three (25%) had a pCR and another 3 (25%) had a near pCR (55). Thus, using chemotherapy and radiation may be an effective modality to improve response rate of locally advanced rectal cancer regardless of their microsatellite status and may be advantageous in MMR-P patients.

## Effectiveness of immunotherapy in rectal cancer patients with MMR-D/MSI-H status

Tumors with MMR-D/MSI-H develop excellent and durable response to CPI due to their high tumor mutation burden (TMB) (56). Preliminary studies with monotherapy or combined CPI have been very encouraging with an excellent clinical response observed among locally advanced colorectal cancer with this biomarker (18, 57–62). In selected studies, surgery was omitted to decrease complication rates linked to the surgical procedure. Cercek et al. (18) reported 12 patients with locally advanced rectal cancer MMR-D who developed a CCR following administration of dostarlimab, an anti-PD-1 antibody, every three weeks for six months. Treatment was well tolerated with no local recurrence at the last follow-up visit. Other studies also corroborated the excellent response rate to CPI for this subset of patients and raised the question whether surgery is needed for local control (59–61). However, those are small studies with a short follow-up. Thus, larger prospective randomized studies are needed to confirm this hypothesis. **Table 3** summarizes response rates of MMR-D/MSI-H colorectal cancer patients to immunotherapy in clinical trials.

**Table 3:** Effectiveness of immunotherapy in colorectal cancer patients with MMR-D/MSI-H status.

Study	Patient No	Immunotherapy	Complete response rate	
			Clinical	Pathological
Cercek et al. (18)	12	Dostarlimab	100%	
Pei et al. (57)	11	Sintilimab		90%
Chalabi et al. (58)	20	Ipilimumab+nivolumab		100%
Xiao et al. (59)	73	PD-1 inhibitor	100%	57%
	(no surgery in 17)			
Zhang et al. (60)	32	PD-1 inhibitor	100%	100%
	(no surgery in 3)			
Yang et al. (61)	20	PD-1 inhibitor	100%	84%
	(no surgery in 7)			
Kothari et al. (62)	9	Pembrolizumab		88%
		Nivolumab		

MMR-D, mismatch repair deficient; MSI-H, microsatellite instability high; No, number.

## Efficacy of immunotherapy among older cancer patients

Older cancer patients tolerate immunotherapy quite well. There was no difference in grade 3-4 toxicity among younger and older cancer patients who were enrolled in phase I clinical trials despite polymedication among the latter group (63). There was no dose reduction among older cancer patients. Other studies also corroborated the safety profile of CPI in older patients with solid tumors. There was no difference in grade 3-5 toxicity in patients 70 years of age and older compared to younger ones (64). However, frail and older cancer patients had more hospital admissions due to a higher comorbidity rate. A meta-analysis of 19 randomized studies of CPI for solid tumors reported improved survival and progression-free survival for both younger and older patients (65). Interestingly, among patients who were 65 years-old or above, the survival magnitude was greater compared to younger ones. Real world data also corroborated the efficacy and safety of CPI in older cancer patients (66). Thus, immunotherapy may be best suited to older cancer patients due to their safety profile.

## The role of radiotherapy for older and frail patients with cancer who are unable to undergo surgery or decline surgery

Surgery has been the main curative treatment for patients with early stage or locally advanced rectal cancers. However, in older patients with multiple comorbidities, surgery may not be feasible due to the high mortality rate and serious complications following surgery (4). Radiotherapy alone or combined with systemic therapy may provide effective palliation, and in selected patients long-term control (67–78).

In early stage rectal cancer (T2 and T3), a high radiation dose delivered by endocavitary contact therapy followed by an iridium implant up to 13,900 cGy has led to a 63% local control rate at 5 years among older patients who were too frail for surgery (70). Radiotherapy alone was well tolerated with minimal complications. The size of the tumor was the main factor affecting survival. The 5-year survival was 84% and 53% for T2 and T3 tumor, respectively. The effectiveness of a high radiation dose delivered with contact therapy or brachytherapy for local control in patients with early stage rectal cancer was also corroborated in another study: CCR and local control were 93% and 72%, respectively (71). Tumor size is again a poor prognostic factor for local control. Thus, a high radiation dose to the cancer is critical for local control and long-term survival in patients with rectal cancer treated with radiotherapy alone.

Radiotherapy is less effective for local control and survival of locally advanced rectal cancer due to a larger tumor mass and a lower radiation dose delivered with external beam radiation either alone or combined with systemic therapy as radiation sensitizer. Clinical complete response rate ranges from 13.5% to 86.2% (67–69, 73–78). Long-term local control and survival have been observed among patients who achieved CCR. Thus, increasing radiation dose to the tumor without damaging the organs at risk (OAR) surrounding the target is critical to achieve a higher rate of CCR and to minimize the risk of serious complications. The addition of brachytherapy as a boost technique following external beam irradiation is an effective technique to spare the OAR as radiation dose decreases exponentially with the distance. As an illustration, high dose rate (HDR) brachytherapy has been reported to be effective to improve local control in rectal cancer patients with a large tumor mass (75). Among 38 patients with locally advanced rectal cancer and a median age of 83 years, 60.6% achieved CCR following hypofractionated external beam irradiation and an HDR boost. Of those with a complete response, 60% were free of disease at two years follow-up. However, 10 patients (26%) developed late complications likely due to the hypofractionation scheme (300 cGy/fraction times 13) and the conventional radiotherapy technique which did not spare the normal tissues from excessive radiation.

Recently, advances in radiotherapy technique such as intensity-modulated radiotherapy (IMRT) and image-guided radiotherapy (IGRT) have allowed clinicians to deliver accurately a high tumor dose while minimizing OAR’s dose, thus improving local control and reducing serious complications in older patients with locally advanced rectal cancer (76). Six patients with locally advanced rectal cancer and a median age of 84 underwent pelvic IMRT to a total dose of, 3900 cGy in 300 cGy/fraction followed by an IGRT HDR boost of, 1200 to, 1800 cGy in 600 cGy/fraction. At a median follow-up of 42 weeks, four patients achieved CCR and were free of disease at the last follow-up visit. No patient developed grade 3-4 complications. This study highlights the importance of modern radiotherapy technique delivery to minimize complications as the radiation dose was similar to the previous study (75). Garant et al. (77) corroborated the safety and efficacy of IGRT to deliver a high tumoricidal radiation dose to older patients with rectal cancer. 94 patients with medically inoperable locally advanced rectal cancer underwent pelvic irradiation to a total dose of, 4000 cGy in 250 cGy/fraction. The residual tumor was boosted with image-guided HDR brachytherapy for an addition dose of, 3000 cGy in three weekly fractions of, 1000 cGy. The CCR and local control rates at 2 years were 86.2% and 71.5%, respectively. 12.8% developed grade 3 bleeding but there was no death related to toxicity.

## The potential role of chemoradiation and immunotherapy to improve response rate in locally advanced rectal cancer and in particular among MMR-P tumor

Another method to improve response rate of locally advanced rectal cancer and to avoid surgery is the combination of radiation sensitizers with hypofractionated external beam radiotherapy. Among 22 patients with inoperable locally advanced rectal cancer, the combination of capecitabine and bevacizumab with a tumor dose of, 5100 in 340 cGy/fraction have led to a 68.5% CCR rate (78). 14 patients (45%) remained disease free at a median follow-up of 18 months. Only two patients (9%) developed fistula likely due to tumor recurrence (n=1) or tumor regression following invasion of the bladder and vagina (n=1). Combining chemotherapy with radiotherapy delivered to a higher dose with brachytherapy may improve further the CCR rate and local control. The proof of this concept was highlighted by the OPERA randomized trial (10). 141 patients with clinical stage T2 or T3 underwent neoadjuvant chemotherapy with oral capecitabine and external beam pelvic irradiation to a total dose of, 4500 cGy. The residual tumor was boosted with either external beam radiation to 900 cGy in five fractions or with endocavitary contact therapy to, 9000 cGy in three fractions. Among patients with larger tumor (3 cm or above) and smaller tumor (<3 cm), the 3-year organ preservation rate was 55% and 68%, respectively. Thus, tumor size is still a prognostic factor for local control in patients who received chemoradiation. As capecitabine and pelvic irradiation may be well tolerated in older and frail cancer patients, chemoradiation should be considered among patients with MMR-P tumors due to their potential to increase PD-L1 expression (52, 79, 80). Advanced techniques of radiotherapy such as IGRT has also been reported to improve tolerance of older rectal cancer patients to chemotherapy and radiotherapy (81). Thus, capecitabine combined with IMRT/IGRT followed by an endocavitary boost through contact therapy or HDR may be a good option for those patients.

Among patients who are physically fit, immunotherapy combined with chemoradiation may further improve the response rate of locally advanced rectal cancer due the synergy of those two modalities (53–55). However, serious toxicity may also increase, thus limiting its efficacy in older and frail rectal cancer patients. Therefore, for the management of older patients with locally advanced rectal cancer, many factors need to be taken into consideration weighing treatment efficacy versus patient tolerance but chronological age alone should not be used to discriminate against those patients.

## Evaluation of frailty in older patients with cancer

As people get older, there is a decrease in the reserve in the body capacity secondary to alteration of the cellular enzymatic and DNA repair system which decreases the body response to stressors, resulting in adverse outcome (82, 83). In frail cancer patients, there is an increased mortality risk with surgery and chemotherapy (4, 84). Older cancer patients (65 years-old or above) should be evaluated for frailty before undergoing any treatment. Even though there are many questionnaires for frailty evaluation, the G-8 questionnaire is simple to administer in a busy clinic (85). Those with a score of 15 or above will be defined as fit. Those with a score of 14 or less will undergo a complete geriatric assessment with the comprehensive geriatric assessment (CG survey (86). We propose a protocol using patient fitness and biomarkers to stratify treatment of older patients with locally advanced rectal cancers who cannot undergo surgery or decline surgery.

## Proposed IGRG algorithm for older patients with locally advanced rectal cancer

All tumor biopsy specimen should undergo next generation sequencing (NGS) if feasible which includes PD-L1 and MMR/MSI status. All patients with MMR-D/MSI-H should be candidates for CPI alone to minimize toxicity. Among patients with MMR-P/MSS, fit patients should undergo chemoradiotherapy and immunotherapy for a better response. Frail patients with positive PD-L1 (1% or more) should receive immunotherapy followed by radiotherapy. An alternative would be to consider immunotherapy and chemotherapy for a better response. However, due to their frailty status, chemotherapy may increase the treatment toxicity and may be best avoided (84). Elderly frail cancer patients have been shown to experience increased grade 3-4 toxicity, frequent hospitalizations and emergency room visits compared to fit patients. Those who are negative PD-L1 (<1%) should receive radiotherapy first to induce upregulation of PD-L1 followed by immunotherapy.

External beam pelvic irradiation should be performed with IMRT and IGRT to minimize complication rates followed by a endocavitary boost with contact therapy or brachytherapy if feasible to deliver a high dose to the residual tumor. However, if the endocavitary boost is not available, IGRT boost with external beam radiation is also a consideration. In frail patients with limited mobility, hypofractionated radiotherapy should be considered to decrease the need for transportation. **Table 4** summarizes the proposed algorithm.

**Table 4:** Proposed algorithm by the International Geriatric Radiotherapy for the management of older patients with locally advanced rectal cancer who are too frail to undergo surgery or who decline surgery.

Biomarker status	Clinical status	
	Fit	Frail
MMR-D/MSI-H	Immunotherapy	Immunotherapy
MMR-P/MSS	ChemoRT+Immunotherapy	RT+Immunotherapy

MMR-D, mismatch repair deficient; MSI-H, microsatellite instability high; MMR-P, mismatch repair proficient; MSS, microsatellite stable; chemoRT, chemoradiation.

Clinicians should be flexible in the management of older cancer patients until data from prospective studies become available. With a network of, 1280 cancer institutions across the world and a large number of patients from all ethnicities, the IGRG is committed to conduct those studies when funding becomes available (87, 88).

## Conclusion

The combination of radiotherapy and immunotherapy may be beneficial for older patients with locally advanced rectal cancer who may be too frail to undergo surgery or who decline surgery to improve the clinical response rate. Prospective studies should be conducted to verify this hypothesis.

## Data availability statement

The original contributions presented in the study are included in the article/supplementary material. Further inquiries can be directed to the corresponding author.

## Author contributions

NN: Writing – original draft, Writing – review & editing. MM: Writing – original draft, Writing – review & editing. AS: Writing – original draft, Writing – review & editing. BP: Writing – original draft, Writing – review & editing. VV-H: Writing – original draft, Writing – review & editing. OG: Writing – original draft, Writing – review & editing. MA: Writing – original draft, Writing – review & editing. TM: Writing – original draft, Writing – review & editing. HG: Writing – original draft, Writing – review & editing. MV: Writing – original draft, Writing – review & editing. FD: Writing – original draft, Writing – review & editing. CT: Writing – original draft. UK: Writing – original draft, Writing – review & editing. ZD: Writing – original draft, Writing – review & editing. EN: Writing – original draft, Writing – review & editing. EL: Writing – original draft, Writing – review & editing. LK: Writing – original draft, Writing – review & editing. JO: Writing – original draft, Writing – review & editing. EO: Writing – original draft, Writing – review & editing. SB: Writing – original draft, Writing – review & editing. TV: Writing – original draft, Writing – review & editing.
